# The Early-Acting Peroxin PEX19 Is Redundantly Encoded, Farnesylated, and Essential for Viability in *Arabidopsis thaliana*

**DOI:** 10.1371/journal.pone.0148335

**Published:** 2016-01-29

**Authors:** Margaret M. McDonnell, Sarah E. Burkhart, Jerrad M. Stoddard, Zachary J. Wright, Lucia C. Strader, Bonnie Bartel

**Affiliations:** 1 Department of BioSciences, Rice University, Houston, Texas, United States of America; 2 Department of Biology, Washington University at St. Louis, St. Louis, Missouri, United States of America; Iwate University, JAPAN

## Abstract

Peroxisomes are single-membrane bound organelles that are essential for normal development in plants and animals. In mammals and yeast, the peroxin (PEX) proteins PEX3 and PEX19 facilitate the early steps of peroxisome membrane protein (PMP) insertion and pre-peroxisome budding from the endoplasmic reticulum. The PEX3 membrane protein acts as a docking site for PEX19, a cytosolic chaperone for PMPs that delivers PMPs to the endoplasmic reticulum or peroxisomal membrane. PEX19 is farnesylated in yeast and mammals, and we used immunoblotting with prenylation mutants to show that PEX19 also is fully farnesylated in wild-type *Arabidopsis thaliana* plants. We examined insertional alleles disrupting either of the two *Arabidopsis* PEX19 isoforms, PEX19A or PEX19B, and detected similar levels of PEX19 protein in the *pex19a-1* mutant and wild type; however, PEX19 protein was nearly undetectable in the *pex19b-1* mutant. Despite the reduction in PEX19 levels in *pex19b-1*, both *pex19a-1* and *pex19b-1* single mutants lacked notable peroxisomal β-oxidation defects and displayed normal levels and localization of peroxisomal matrix and membrane proteins. The *pex19a-1 pex19b-1* double mutant was embryo lethal, indicating a redundantly encoded critical role for PEX19 during embryogenesis. Expressing YFP-tagged versions of either PEX19 isoform rescued this lethality, confirming that PEX19A and PEX19B act redundantly in *Arabidopsis*. We observed that *pex19b-1* enhanced peroxisome-related defects of a subset of peroxin-defective mutants, supporting a role for PEX19 in peroxisome function. Together, our data indicate that *Arabidopsis* PEX19 promotes peroxisome function and is essential for viability.

## Introduction

Peroxisomes are eukaryotic organelles that house critical oxidative reactions and sequester harmful reactive oxygen species to prevent damage to other cellular compartments. Peroxisomal enzymes participate in diverse metabolic processes, including photorespiration and fatty acid β-oxidization (reviewed in [1]). Additionally, peroxisomal enzymes convert the auxin precursor indole-3-butyric acid (IBA) to the active auxin indole-3-acetic acid (IAA) by β-oxidization [2–6]; IAA derived from IBA contributes to seedling cell expansion [5, 7] and lateral root production [5, 8, 9]. Dysfunctional plant peroxisomes can cause a variety of physiological defects, including reduced germination, stunted growth, poor fertility, and lethality (reviewed in [1, 10]). Defects in human peroxisomes underlie congenital peroxisome biogenesis disorders that result in a spectrum of dysfunctions that often are fatal (reviewed in [11]).

Peroxin (PEX) proteins function in *de novo* peroxisome biogenesis, division, and matrix protein import. Peroxisome matrix proteins are synthesized in the cytosol and usually are targeted to the peroxisome by a carboxyl-terminal three-amino acid peroxisome-targeting signal 1 (PTS1), which is recognized by the cytosolic PTS1 receptor, PEX5 [12]. A less common targeting mechanism uses an N-terminal nine-amino acid PTS2, which is recognized by the cytosolic PTS2 receptor, PEX7 [13, 14]. Cargo-bound PEX5 and PEX7 associate with the docking proteins, PEX13 and PEX14, residing in the peroxisome membrane (reviewed in [15]). PEX5 forms part of a transient pore that facilitates cargo entry into the peroxisome [16], after which PEX5 is recycled with the assistance of PEX4, a ubiquitin-conjugating enzyme, and the ubiquitin-protein ligases PEX2, PEX10, and PEX12 (reviewed in [15]). Ubiquitinated PEX5 is retrotranslocated out of the peroxisome by the PEX1 and PEX6 ATPases [15], deubiquitinated, and released into the cytosol to be used for additional import. Inefficiently retrotranslocated PEX5 can be poly-ubiquitinated and degraded by the proteasome [15].

Peroxisomes can multiply by fission of pre-existing peroxisomes and by budding from the endoplasmic reticulum (ER; reviewed in [1]). Three peroxins, PEX19, PEX3, and PEX16 are implicated in *de novo* biogenesis of peroxisomes (reviewed in [17]). In mammals and plants, PEX16 resides in the ER membrane and recruits PEX3 [18, 19], which in turn docks PEX19 [20]. Some organisms, such as *Saccharomyces cerevisiae*, lack PEX16 and PEX3 appears to target to the ER directly (reviewed in [21]). Budding of pre-peroxisomes from the ER and PMP insertion require PEX3 and PEX19 in yeast [22]; *pex3* and *pex19* mutants appear to lack peroxisomes [23, 24]. PEX19 acts as a chaperone for peroxisome membrane proteins (PMPs), binding PMPs near their transmembrane domains [25] and allowing transfer to PEX3 and insertion in the membrane [26, 27]. PEX19 promotes PMP targeting to peroxisomes in human fibroblasts [28]; nuclear localization of PEX19 results in mislocalization of PMPs to the nucleus [29].

The PEX19 C-terminus is farnesylated in yeast [23] and mammals [30]; this post-translational modification increases the strength of PEX19-PMP interactions [31, 32], suggesting that the farnesyl moiety might assist peroxisome biogenesis. Although farnesylation can promote membrane association of some proteins (reviewed in [33]), PEX19 is cytosolic in yeast [34], humans [29], and plants [35].

Although plants have homologs of the three early-acting peroxins [36], neither *pex3* nor *pex19* mutants has emerged from forward-genetic screens for mutants with defective peroxisome (reviewed in [10]), perhaps because PEX3 and PEX19 each have two isoforms in *Arabidopsis*. However, *Arabidopsis* RNAi lines targeting *PEX3*, *PEX16*, or *PEX19* have enlarged peroxisomes that display reduced matrix protein import [37], confirming a role in plant peroxisome biology for the early-acting peroxin homologs. Moreover, *Arabidopsis* PEX19 binds to PEX10 [35] and PXA1 [38] *in vitro*, consistent with a PMP chaperone function. In this work, we explore the roles of PEX19 in *Arabidopsis* and reveal that *Arabidopsis* PEX19 is redundantly encoded, farnesylated, and essential for embryogenesis.

## Materials and Methods

### Plant materials and growth conditions

*Arabidopsis thaliana* accession Columbia (Col-0) was used as wild type. *pex19a-1* (SALK_020100), *pex19b-1* (SAIL_76_C06), *ggb-3* (SALK_015072), and *plp-4* (GABI-KAT 386C07) were obtained from the *Arabidopsis* Biological Resource Center (Ohio State University). *era1-2* [39], *pex2-1* [40], *pex7-2* [41], *pex10-2* [40], *pex13-4* [42], and *pex14-2* [43] were previously described. Mutations were followed in segregating populations by using PCR-based genotyping (S1 Table) and antibiotic resistance.

Seeds were surface-sterilized with 30% (v/v) commercial bleach, 0.01% (v/v) Triton X-100 and stratified for 1–3 days at 4°C in 0.1% agar. For experiments that included *era1-2*, 10 μM gibberellin (GA_3_) was included in the stratification medium for all lines. Stratified seeds were plated on plant nutrient (PN) medium [44] or on PN supplemented with 0.5% sucrose (PNS), with or without IBA. IBA was dissolved in ethanol at 100 mM and control media were normalized to the same ethanol content. Seedlings transferred from plates to soil were grown at 22°C under continuous illumination.

For assays of light-grown seedlings, seeds were stratified for 1–3 days and plated on the indicated media. After 8 days of growth at 22°C under continuous light filtered through yellow long-pass filters, which slow the breakdown of indolic compounds [45], seedling roots were measured. For assays of dark-grown seedlings, seeds were stratified for 1–2 days, plated on the indicated media, placed under yellow light for one day, and then placed in darkness for 4 days. Hypocotyls of germinated seedlings were measured following the 4-day dark period.

### Statistical analysis

One-way ANOVA analysis followed by Duncan’s test was performed using the SPSS Statistics software program (version 22.0.0.1). For each treatment condition (e.g., no sucrose, IBA), mean root or hypocotyl lengths that were not significantly (*P* < 0.001) different from each other are designated with the same letter above the bar.

### PEX19 fusion protein expression

The *PEX19A* (G66139) and *PEX19B* (G13403) cDNAs were obtained from the *Arabidopsis* Biological Resource Center (Ohio State University) and recombined into the pEG104 (N-terminal YFP tag, driven by the cauliflower mosaic virus *35S* promoter) and pEG201 (N-terminal HA tag, driven by the cauliflower mosaic virus *35S* promoter) vectors [46] using LR Clonase (Invitrogen) to form *35S*:*YFP-PEX19A*, *35S*:*YFP-PEX19B*, *35S*:*HA-PEX19A*, and *35S*:*HA-PEX19B*. Plasmids were electroporated into *Agrobacterium tumefaciens* GV3101 (pMP90) [47] and used to transform wild-type Col-0 using the floral dip method [48]; transformants were selected on 7.5 μg/mL glufosinate ammonium (Basta). Homozygous lines were obtained from the progeny of transformants by following Basta resistance in subsequent generations.

To obtain *pex19a-1 pex19b-1 35S*:*YFP-PEX19A* and *pex19a-1 pex19b-1 35S*:*YFP-PEX19B*, we crossed a *PEX19A/pex19a-1 pex19b-1*/*pex19b-1* plant to Col-0 transformed with *35S*:*YFP-PEX19A* or *35S*:*YFP-PEX19B*. Lines homozygous for both *pex19* mutations and the YFP transgene were isolated using PCR-based genotyping and confirmed using resistance to kanamycin (conferred by the *pex19a-1* T-DNA), YFP fluorescence, and immunoblotting.

### Immunoblot analysis

Protein was extracted by grinding frozen tissue and adding two volumes of 2x sample buffer (Invitrogen, Carlsbad, CA). Samples were centrifuged, and a 15 μL aliquot of supernatant was transferred to a new tube with 1.6 μL of 0.5 M dithiothreitol and heated at 100°C for 5 minutes. Samples were loaded in 10% or 12% NuPAGE Bis-Tris gels (Invitrogen) next to prestained protein markers (P7708S, New England Biolabs, Beverly, MA) and Cruz Markers (Santa Cruz Biotechnology, Santa Cruz, CA). After electrophoresis using NuPAGE MOPS-SDS running buffer (Invitrogen), proteins were transferred for 45 minutes at 24 V to a Hybond ECL nitrocellulose membrane (Amersham Pharmacia Biotech, Piscataway, NJ) using NuPAGE transfer buffer (Invitrogen). After transfer, membranes were rocked for 1 hour at 4°C in blocking buffer (8% non-fat dry milk [w/v], 20 mM Tris, pH 7.5, 150 mM NaCl, 0.1% Tween-20) and incubated overnight at 4°C with primary antibodies diluted in blocking buffer: 1:1,000 rabbit α-APX3 [49, 50], 1:100 rabbit α-GFP (Clontech 632376), 1:100 rabbit α-PEX5 [51], 1:800 rabbit α-PEX7 [41], 1:500 rabbit α-PEX10 [40], 1:10,000 rabbit α-PEX14 (Agrisera AS08 372), 1:500 or 1:2000 rabbit α-PEX19 (generated and affinity purified by Proteintech Group using a recombinant protein that included the full amino acid sequence of PEX19B), 1:2,000 rabbit α-PMDH2 [52], 1:5,000 rabbit α-thiolase [53], 1:50,000 or 1:100,000 mouse α-HSC70 (StressGen Bioreagents SPA-817), or 1:2,000 mouse α-mito ATP synthase (MitoScience MS507). Membranes were rinsed twice with blocking buffer followed by a 4–5 hour incubation with horseradish peroxidase-linked goat α-rabbit or α-mouse IgG secondary antibody (1:5,000; Santa Cruz Biotechnology, SC2030 or SC2031). After rinsing in 20 mM Tris, pH 7.5, 150 mM NaCl, 0.1% Tween-20, horseradish peroxidase activity was visualized using WesternBright ECL reagent (Advansta, Menlo Park, CA) and exposure to autoradiography film. Membranes were reblocked with blocking buffer and sequentially probed with additional indicated antibodies.

### Confocal microscopy

The import of PTS2 proteins was observed using plants expressing *35S*:*PTS2-GFP*, which encodes a GFP extended with the N-terminal 49 amino acid residues from the PED1 isoform of thiolase [54]. Fluorescence in plants expressing *35S*:*YFP-PEX19A* and *35S*:*YFP-PEX19B* were compared to plants expressing *35S*:*YFP-ECH2*, which marks the peroxisome matrix [5], *35S*:*ER-YFP-HDEL* (ER-yk), which marks the ER lumen [55], and *35S*:*YFP* [46], which marks the cytoplasm. For confocal imaging, cotyledons from 5-day-old light-grown seedlings were mounted in water under a cover slip. Images of epidermal cells were collected using a Carl Zeiss LSM 710 laser scanning confocal microscope equipped with a Meta detector. GFP samples imaged through a 40x oil immersion objective were excited with a 488-nm argon laser; emission was collected between 494 and 560 nm. Each image averaged 8 exposures using a 23-μm pinhole corresponding to a 0.7 μm optical slice. YFP samples imaged through a 63x oil immersion objective were excited with a 488-nm argon laser; emission was collected between 493 and 555 nm. Each image averaged 4 exposures using a 44.8-μm pinhole corresponding to a 0.8 μm optical slice.

### Fractionation

Fractionation of seedling extracts into organellar and cytosolic fractions was modified from Burkhart et al., 2014. Seeds (1 mg) were plated on PNS medium and incubated under white light for 5 days. Seedlings were minced with scissors in 1 mL ice-cold fractionation buffer [150 mM Tris pH 7.6, 10 mM KCl, 1 mM EDTA, 1 mM DTT, 100 mM sucrose, 1 mM PMSF, 1 mM NEM, 1x plant protease inhibitor cocktail (Sigma P9599)]. Minced samples were transferred to a 1 mL Dounce homogenizer, homogenized for 20 strokes, and filtered through Miracloth (Millipore). Samples were centrifuged for 10 minutes at 640 rpm at 4°C to pellet unlysed cells, giving the homogenate fraction. Homogenate (200 μL) was centrifuged at 12,000 rpm for 20 minutes at 4°C, giving the supernatant fraction. The pellet was washed once with 200 μL fractionation buffer and centrifuged at 12,000 rpm for 20 minutes at 4°C, giving the wash fraction. The pellet was resuspended in fractionation buffer equal to the homogenate volume, giving the pellet fraction. Following fractionation, an aliquot of each fraction was added to equal volume of NuPAGE 2x sample buffer (Invitrogen), and 15 μL of each sample was processed for immunoblotting.

## Results

### PEX19 is encoded by two genes in *Arabidopsis*

**Fig 1:**
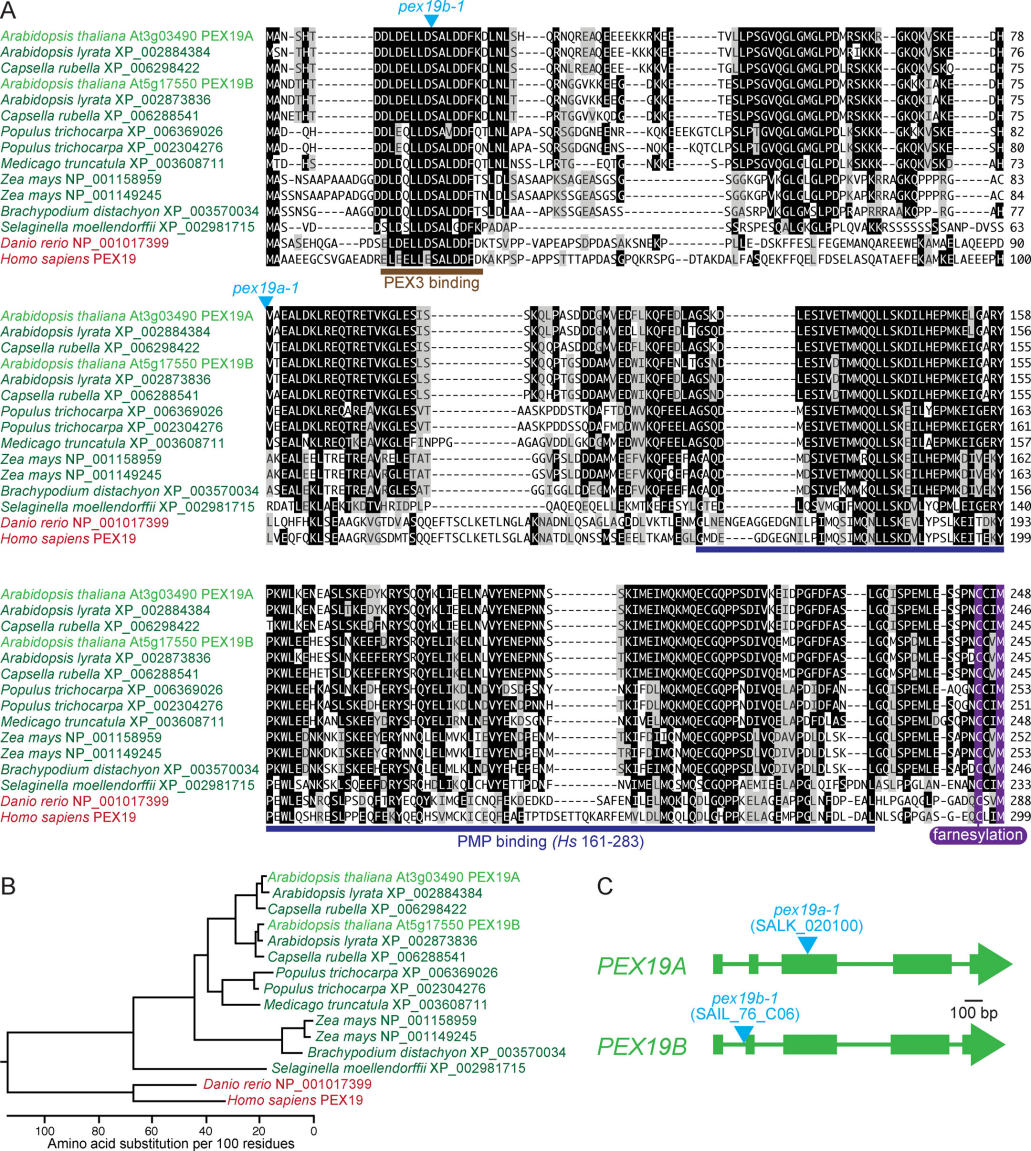
PEX19 is encoded by two genes in *Arabidopsis*. (A) Alignment of PEX19A and PEX19B from various plants (green) with the zebra fish (*Danio rerio*) and human (*Homo sapiens*) homologs (red), highlighting the carboxyl-terminal CaaM farnesylation motif (purple) and the domains implicated in PEX3 (brown) and PMP (blue) binding in human PEX19 [56–58]. Sequences were aligned using MegAlign program (DNAStar) and the Clustal W method. Residues identical in at least seven sequences are boxed in black, chemically similar residues are boxed in gray. The sites of the T-DNA insertions in the *pex19a-1* and *pex19b-1* are indicated by triangles above the sequences. (B) Phylogenetic tree showing relationships of proteins in panel A generated by the MegAlign program. The *Arabidopsis* PEX19A and PEX19B duplication is found in closely related plants, such as *Arabidopsis lyrata* and *Capsella rubella*, but not in more distantly related plants, such as *Medicago* or *Brachypodium*. (C) *PEX19A* and *PEX19B* gene diagrams showing T-DNA insertion sites with triangles, introns as lines, and exons as boxes.

There are two isoforms of PEX19 in *Arabidopsis thaliana* [35] and closely related plants (Fig 1A). Although PEX19 duplications appear to have occurred more than once in the plant lineage, some plants carry only a single *PEX19* gene (Fig 1B). The two *Arabidopsis PEX19* genes were initially named *AtPEX19-1* (At3g03490) and *AtPEX19-2* (At5g17550) [35]. For clarity in describing mutant alleles, in this work we refer to *AtPEX19-1* as *PEX19A* and *AtPEX19-2* as *PEX19B*. PEX19A and PEX19B are 84% identical at the amino acid level (Fig 1A) and are encoded by mRNAs that are 79% identical at the nucleotide level. To characterize the *in vivo* roles of the two PEX19 isoforms, we obtained two T-DNA alleles, one containing an insert in the third exon of *PEX19A* (SALK_020100), which we named *pex19a-1* (Fig 1C), and one containing an insert in the first intron of *PEX19B* (SAIL_76_C06), which we named *pex19b-1* (Fig 1C).

### *Arabidopsis* PEX19 is farnesylated

**Fig 2:**
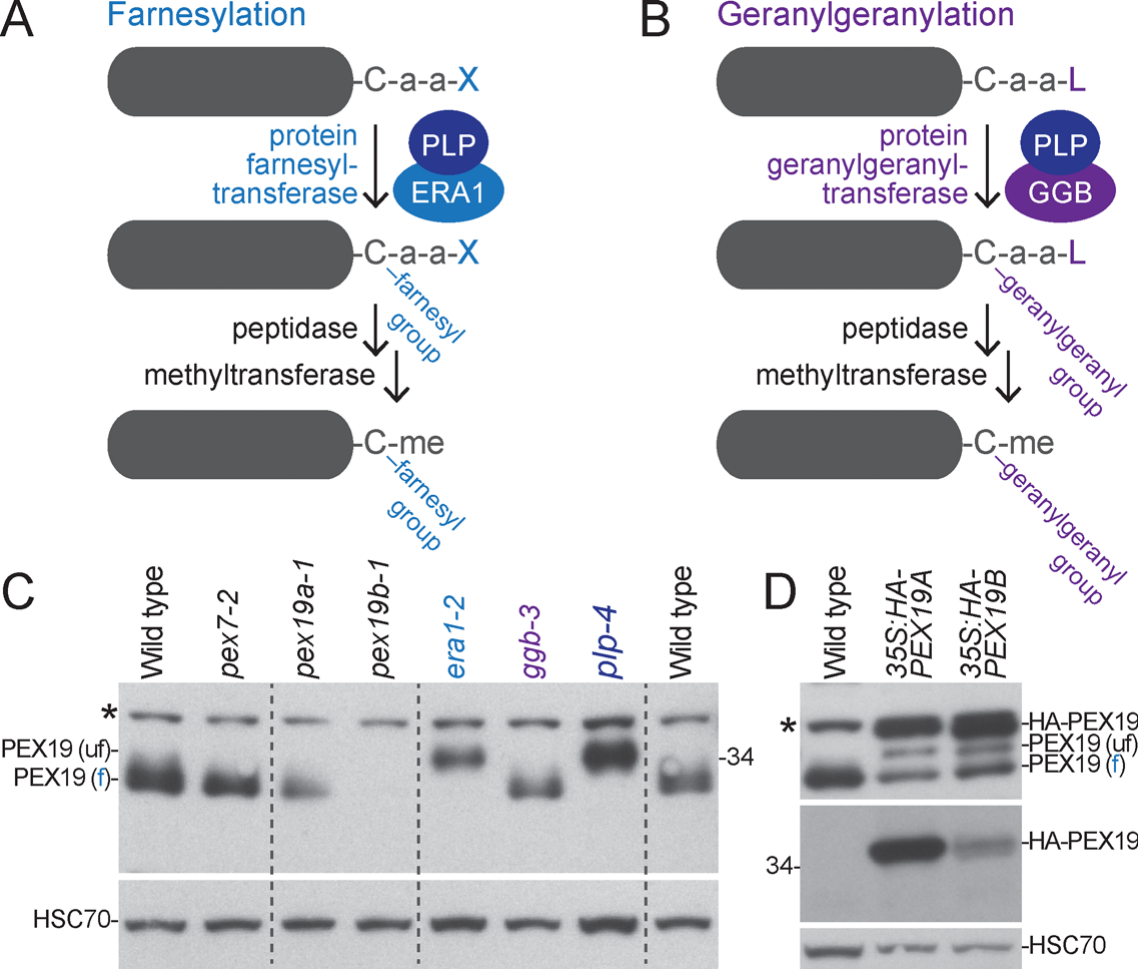
*Arabidopsis* PEX19 is farnesylated. (A) Proteins with a C-terminal CaaX motif (Cys-aliphatic-aliphatic-X; where X can be Ser, Met, Ala, Asn, or Cys) can be farnesylated by a protein farnesyl-transferase complex composed of PLP and ERA1, cleaved of the three carboxyl-terminal residues, and methylated (me) on the carboxyl group of the prenylated Cys residue [59]. (B) Proteins with a C-terminal a CaaL motif (Cys-aliphatic-aliphatic-Leu) can be geranylgeranylated by protein geranylgeranyl-transferase complex composed of PLP and GGB [59]. (C) PEX19 is farnesylated *in vivo*. Protein from 8-day-old light-grown seedlings was separated using 12% PAGE and processed for immunoblotting with antibodies recognizing PEX19 and HSC70 (loading control). The positions of the molecular mass markers (in kDa) are indicted at the right. The positions of unfarnesylated (u) and farnesylated (f) PEX19 are indicated at the left. An asterisk marks a protein that cross-reacts with the PEX19 antibody. (D) HA-PEX19 expression decreases farnesylation of endogenous PEX19. Protein extracted from 4-day-old light-grown seedlings was separated using 12% PAGE and processed for immunoblotting with antibodies recognizing PEX19 (top panel), the HA epitope (middle panel), and HSC70 (bottom panel; loading control). The positions of the molecular mass markers (in kDa) are indicted at the left. The positions of unfarnesylated (u), farnesylated (f), and HA-tagged PEX19 are indicated at the right. An asterisk marks a protein that cross-reacts with the PEX19 antibody.

PEX19 is farnesylated in yeast and mammals [30, 32]. Farnesylation is a post-translational modification in which a 15-carbon hydrophobic moiety is attached to the Cys (C) residue in the carboxyl-terminal CaaX motif, where “a” is an aliphatic residue and “X” is Ser, Met, Ala, Gln, or Cys (reviewed in [33, 59]; Fig 2A). Similarly, geranylgeranylation attaches a 20-carbon hydrophobic moiety to the Cys residue of a carboxyl-terminal CaaL (Fig 2B). Both prenylation variants can facilitate protein-membrane or protein-protein interactions [33]. The conservation of a PEX19 farnesylation motif (CaaM) across several distantly related organisms (Fig 1A) suggests that this sequence is important for PEX19 function.

To determine if PEX19 is farnesylated in *Arabidopsis*, we generated an antibody to PEX19B and used immunoblotting to examine protein extracts prepared from three prenylation-defective mutants. We assayed a mutant (*era1-2*) with a fast-neutron induced deletion of the *ENHANCED RESPONSE TO ABSCISIC ACID* gene, which encodes the β-subunit of protein farnesyltransferase (Fig 2A) [39], a mutant (*ggb-3*) carrying a T-DNA insertion in the *GERANYLGERANYLTRANSFERASE BETA* (*GGB*) gene, which encodes the β-subunit of protein geranylgeranyltransferase (Fig 2B) [60], and a mutant (*plp-4*) disrupted in both types of prenylation because of a T-DNA insertion in the *PLURIPETALA* gene, which encodes the common α-subunit of both prenylation enzymes (Fig 2A and 2B) [61]. The anti-PEX19 antibody detected an approximately 30-kDa protein in wild-type seedling extracts that migrated more slowly in the *plp-4* mutant (Fig 2C), implying that PEX19 is fully prenylated in wild type and that prenylation increases the electrophoretic mobility of the PEX19 protein. We also observed the more slowly migrating form of PEX19 in the *era1-2* mutant, implying that PEX19 is largely farnesylated in wild-type *Arabidopsis* (Fig 2C). In contrast, PEX19 migration resembled wild type in *ggb-3* (Fig 2C), indicating that *Arabidopsis* PEX19 is not appreciably geranylgeranylated. We concluded that *Arabidopsis* PEX19 is farnesylated *in vivo* through the action of ERA1 and PLP.

To determine whether our PEX19B antibody also detected PEX19A, we expressed N-terminally HA-tagged PEX19A or PEX19B from the *35S* cauliflower mosaic virus promoter. Both lines expressed PEX19 protein that was detected by an anti-HA antibody and by our anti-PEX19B antibody (Fig 2D), indicating that our antibody detected both PEX19A and PEX19B. The anti-HA antibody revealed that the *35S*:*HA-PEX19A* line accumulated more HA-PEX19 than did the *35S*:*HA-PEX19B* line (Fig 2D). The apparently similar HA-PEX19 levels in the two lines detected by our anti-PEX19B antibody (Fig 2D) indicate that our antibody detects PEX19B more effectively than PEX19A. Interestingly, expression of HA-PEX19A or HA-PEX19B appeared to reduce farnesylation of native PEX19, as bands migrating with the mobility of both unfarnesylated and farnesylated PEX19 were detected in the *35S*:*HA-PEX19* lines (Fig 2D). This finding implies that the farnesylation machinery can be overwhelmed by PEX19 overexpression.

### An insertional allele disrupting *PEX19B* accumulates reduced levels of PEX19 protein

To examine the functions of *Arabidopsis* PEX19, we isolated mutants carrying disrupted *PEX19A* or *PEX19B* alleles (Fig 1C) from publically available T-DNA insertion collections [62]. We found that PEX19 levels were dramatically reduced in 8-day-old seedlings of the *pex19b-1* mutant (Fig 2C), which has a T-DNA inserted in the first intron of *PEX19B* (Fig 1C). In contrast, the *pex19a-1* mutant, which has a T-DNA in the third exon of *PEX19A* (Fig 1C), appeared to accumulate normal levels of PEX19 protein (Fig 2C). This analysis suggested that PEX19B is more abundant in seedlings than PEX19A.

**Fig 3:**
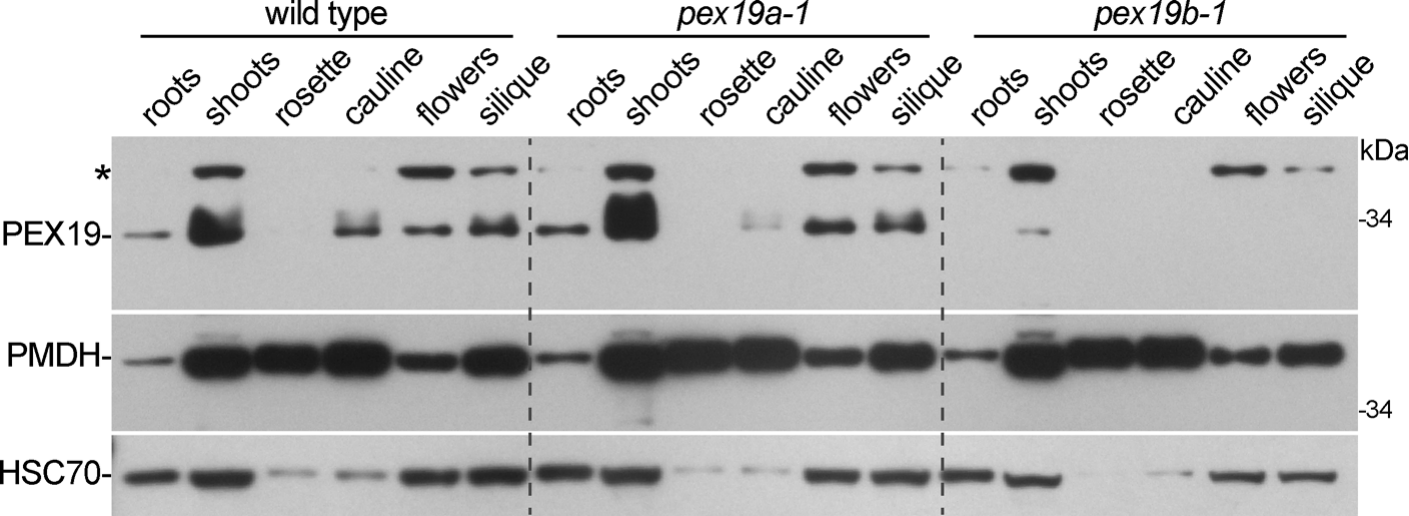
The *pex19b-1* mutant lacks detectable PEX19 protein in various tissues. 8-day-old wild-type, *pex19a-1*, and *pex19b-1* seedlings were separated into roots and aerial tissues (shoots); other tissues were collected from 31-day-old plants: rosette leaf (beginning to senesce), oldest cauline leaf, open flowers, and green siliques (third elongated silique from the apex). Extracts were separated using 10% PAGE and processed for immunoblotting with antibodies recognizing PEX19, PMDH, and HSC70. The positions of the molecular mass markers (in kDa) are indicted at the right. An asterisk marks a protein that cross-reacts with the PEX19 antibody.

To determine where PEX19A and PEX19B proteins accumulated, we compared PEX19 levels in various tissues from *pex19a-1* to detect PEX19B and tissues from *pex19b-1* to detect PEX19A. We examined PEX19 levels in roots and shoots from 8-day-old seedlings and from rosette leaves, cauline leaves, flowers, and green siliques from 31-day-old plants. In wild-type plants, PEX19 protein was detected in all examined tissues except mature rosette leaves, with highest accumulation in seedling shoots (Fig 3). PEX19 protein accumulation resembled wild type in the *pex19a-1* mutant (Fig 3). In *pex19b-1*, we only detected PEX19 protein in seedlings aerial tissues, suggesting that PEX19B is the predominant isoform in many tissues but that PEX19A may function along with PEX19B in seedling aerial tissues. However, because our antibody detects PEX19A less effectively than PEX19B (Fig 2D), we cannot rule out the possibility that PEX19A is present in tissues in addition to seedling shoots. We compared PEX19 protein accumulation patterns to peroxisomal malate dehydrogenase (PMDH) and found that PMDH accumulated more uniformly than PEX19 in various aerial tissues (Fig 3). The relatively higher PEX19 to PMDH ratio in seedlings versus mature leaves (Fig 3) suggests that PEX19, and perhaps peroxisome biogenesis, might be more important in rapidly growing leaves than in mature leaves.

### *pex19* single mutants lack marked peroxisome-associated defects

**Fig 4:**
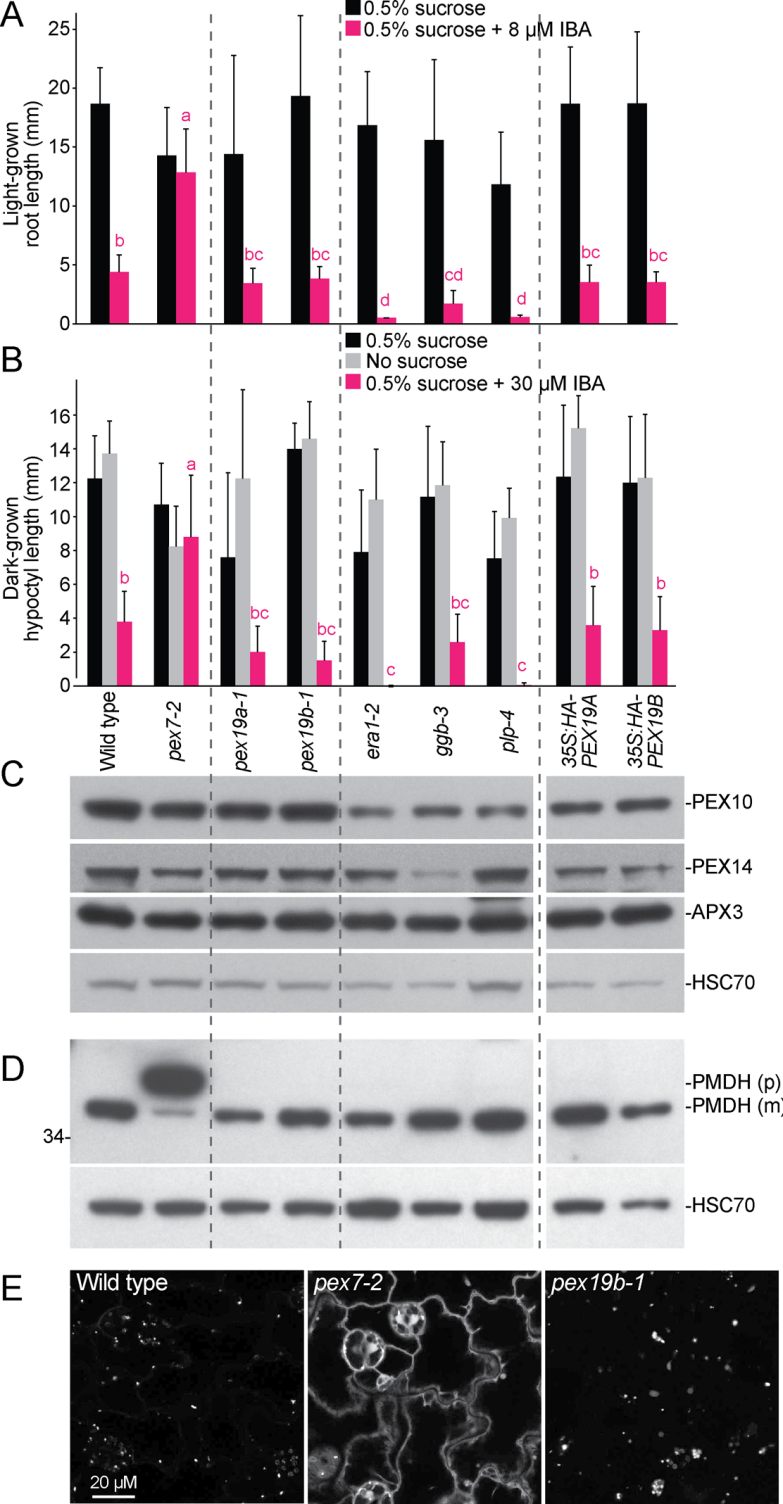
PEX19 farnesylation is not required to maintain peroxisome function. (A) Light-grown *pex19a-1*, *pex19b-1*, and seedlings expressing HA-PEX19 display wild-type IBA sensitivity. The *era1-2*, *ggb-3*, and *plp-4* prenylation mutants also are IBA sensitive. *pex7-2* is an IBA-resistant control. Error bars show standard deviations of mean 8-day-old root lengths (*n* ≥ 7). Different letters above bars indicate significantly different means (one-way ANOVA, *P* < 0.001). (B) Dark-grown *pex19a-1*, *pex19b-1*, and seedlings expressing HA-PEX19 display wild-type IBA sensitivity and sucrose independence. The prenylation mutants are also IBA sensitive and sucrose independent. *pex7-2* is an IBA-resistant control. Error bars show standard deviations of mean 5-day-old hypocotyl lengths (*n* ≥ 8). Different letters above bars indicate significantly different means (one-way ANOVA, *P* < 0.001). (C) *pex19a-1*, *pex19b-1*, prenylation mutants, and seedlings expressing HA-PEX19 display wild-type levels of several PMPs. Protein extracted from 4-day-old light-grown seedlings was separated using 10% PAGE and processed for immunoblotting. The membrane was serially probed with antibodies recognizing the indicated proteins. (D) *pex19a-1*, *pex19b-1*, prenylation mutants, and wild-type seedlings expressing HA-PEX19 fully process the PTS2 region of PMDH. Protein from 8-day-old light-grown seedlings was separated using 10% PAGE and processed for immunoblotting with antibodies recognizing PMDH or HSC70 (loading control). The positions of the molecular mass markers (in kDa) are indicted at the left. PMDH is synthesized as a precursor (p) with a cleavable PTS2 signal that is processed into the mature (m) protein in the peroxisome; this processing is impaired in the *pex7-2* mutant. (E) The *pex19b-1* mutant displays normal import of peroxisomally-targeted GFP. GFP fluorescence of cotyledon epidermal cells from 5-day-old light-grown seedlings carrying the *35S*:*PTS2-GFP* construct was imaged using confocal microscopy.

Plant peroxisomes house enzymes catalyzing β-oxidation of fatty acids (reviewed in [63]) and conversion of indole-3-butyric acid (IBA) to the active auxin indole-3-acetic acid (IAA) [2–6]. Consequently, peroxisome-defective mutants often display growth defects that are ameliorated by provision of sucrose and resistance to the inhibitory effects of IBA on root or hypocotyl elongation [2, 40, 43, 51, 54, 64, 65]. For example, *pex7-2* [41] displays IBA-resistant root elongation in the light (Fig 4A) and IBA-resistant hypocotyl elongation in the dark (Fig 4B). Unlike typical *pex* mutants, we found that the *pex19a-1* and *pex19b-1* mutants grew normally in the absence of sucrose (Fig 4B) and responded to IBA similarly to wild type (Fig 4A and 4B). Moreover, the *35*:*HA-PEX19A* and *35S*:*HA-PEX19B* lines also resembled wild type in these assays (Fig 4A and 4B), confirming that seedling peroxisome function is not highly sensitive to PEX19 dosage. In addition, we found that the prenylation mutants *era1-2*, *ggb-3*, and *plp-4* were sucrose independent and IBA sensitive (Fig 4A and 4B), indicating that prenylation in general and PEX19 farnesylation in particular are not required for efficient seedling peroxisome function.

We indirectly examined peroxisomal matrix protein import using the PTS2 protein PMDH to determine if the *pex19a-1* or *pex19b-1* displayed compromised PTS2-protein processing. The N-terminal PTS2-containing region is cleaved inside the peroxisome matrix following import, resulting in a molecular mass shift that can be detected by immunoblotting. The *pex7-2* mutant displays a clear PTS2-processing defect [41] (Fig 4D) whereas *pex19a-1*, *pex19b-1*, the prenylation mutants, and the *35*:*HA-PEX19* lines all appeared to process PMDH similarly to wild type in seedlings (Fig 4D), where PEX19 is particularly abundant (Fig 3). Moreover, PMDH processing resembled wild type in various tissues and growth stages of the *pex19* mutants (Fig 3), suggesting efficient import of matrix proteins in the *pex19* mutants at additional developmental time points.

We used confocal microscopy to directly examine PTS2 protein import in *pex19b-1*. Wild-type seedlings expressing *35S*:*PTS2-GFP* [54] displayed the expected punctate pattern, indicating efficient matrix protein import, whereas *pex7-2* displayed extensive cytosolic fluorescence [41] (Fig 4E). In *pex19b-1*, PTS2-GFP fluorescence resembled wild type (Fig 4E), again indicating successful import of PTS2-targeted proteins in this mutant.

PEX19 is implicated in inserting PMPs into membranes, and yeast *pex19* mutants display reduced PMP levels [34]. Therefore, we examined levels of three PMPs, peroxisomal ascorbate peroxidase (APX3) and the membrane peroxins PEX10 and PEX14 in *pex19* mutants. We found that the levels of these PMPs were similar to wild type in *pex19a-1*, *pex19b-1*, the prenylation mutants, and the *35*:*HA-PEX19* lines (Fig 4C), suggesting that altering PEX19 levels or prenylation did not dramatically alter PMP stability in *Arabidopsis*.

**Fig 5:**
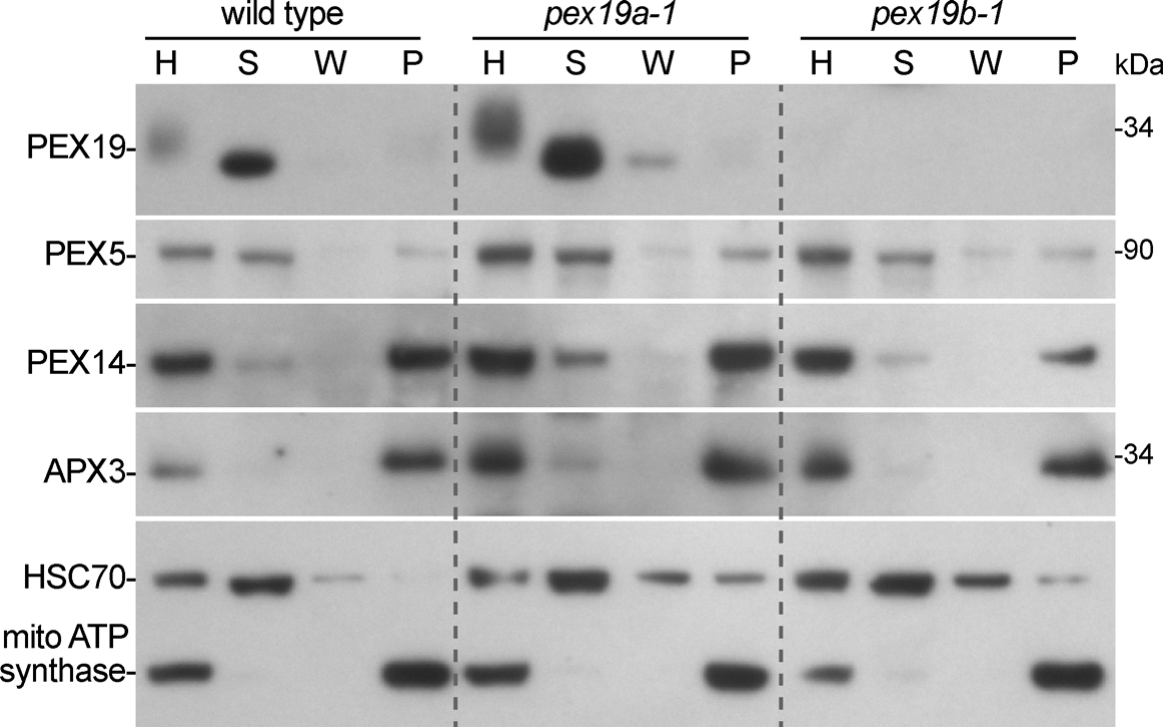
PEX19 is a soluble protein, and PMPs remain membrane-associated in *pex19* mutants. Homogenates (H) from 5-day-old light-grown seedlings were fractionated using centrifugation to give a supernatant (S) containing soluble proteins, a wash (W) fraction, and a pellet (P) containing membrane-associated proteins. Fractions were separated using 10% PAGE and processed for immunoblotting with antibodies recognizing the indicated proteins. The positions of the molecular mass markers (in kDa) are indicted at the right. Antibodies recognizing HSC70 (a cytosolic protein) and mitochondrial ATP synthase (a membrane protein) were used to monitor fractionation.

Because PMP levels were not altered in *pex19* mutants, we examined whether membrane associations of PMPs were altered by using centrifugation to fractionate wild-type and mutant seedling extracts. We found PEX14 and APX3 in the pellet fraction in wild type, *pex19a-1*, and *pex19b-1* (Fig 5), suggesting that these PMPs remain membrane-associated despite the low PEX19 protein levels in the *pex19b-1* mutant. In addition, we detected PEX19 in the soluble fraction in wild-type and *pex19a-1* seedlings (Fig 5), confirming previous reports that PEX19 is cytosolic in *Arabidopsis* [35] as it is in yeast [34] and mammals [29]. Moreover, the peroxisomal matrix protein receptor, PEX5, was distributed similarly between the soluble and pellet fraction of wild type, *pex19a-1*, and *pex19b-1* (Fig 5), suggesting that PEX5 localized normally in *pex19a-1* and *pex19b-1*.

### PEX19 is essential for embryogenesis

Because we did not detect peroxisome-defective phenotypes in either *pex19* single mutant, we explored whether *PEX19A* and *PEX19B* act redundantly by attempting to isolate a *pex19a-1 pex19b-1* double mutant. Although *PEX19A* and *PEX19B* are on different chromosomes, we failed to recover homozygous *pex19a-1 pex19b-1* double mutants from more than 100 F_2_ seedlings from a cross of *pex19a-1* to *pex19b-1*. Therefore, we plated seeds from three *PEX19A/pex19a-1 pex19b-1/pex19b-1* plants and genotyped *PEX19A* in 50 individual seedlings. A homozygous *pex19a-1/pex19a-1 pex19b-1/pex19b-1* double mutant was not isolated. The genotype ratios were consistent with the hypothesis that the *pex19a-1 pex19b-1* double mutant is embryo lethal (Table 1).

**Table 1:** Progeny of *PEX19A/pex19a-1 pex19b-1/pex19b-1*.

*PEX19A*	*PEX19B*	Observed (*n* = 50)	Expected if double mutant is not viable (*n* = 50)	Expected if double mutant is viable (*n* = 50)
+/+	-/-	11	17	12.5
+/-	-/-	39	33	25
-/-	-/-	0	0	12.5

χ^2^ = 20.52, 2 degrees of freedom. P = 0.0001

**Fig 6:**
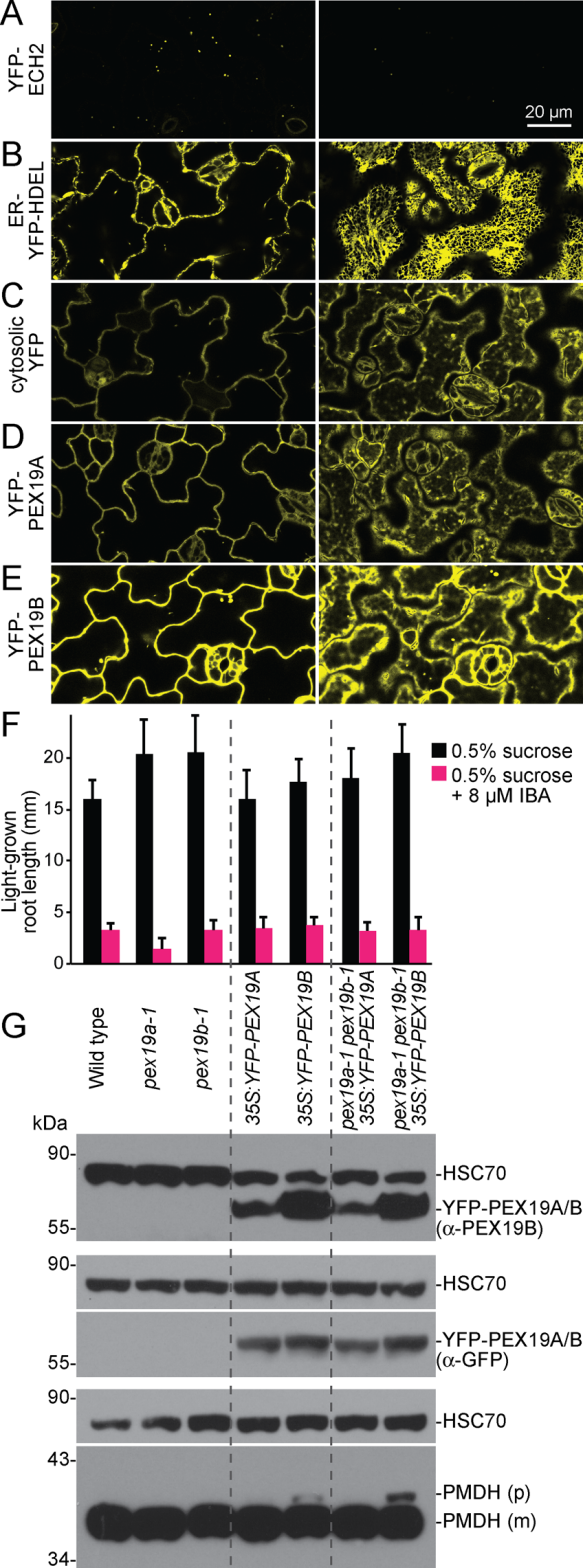
YFP-PEX19 is cytosolic and rescues the embryo lethality of the *pex19a-1 pex19b-1* double mutant. (A-E) YFP-PEX19 is mostly cytosolic. Cotyledon epidermal cells from 5-day-old light-grown seedlings carrying various YFP-tagged constructs were imaged using confocal microscopy. YFP directed to the peroxisome (YFP-ECH2) displays punctate fluorescence (A) and ER-directed YFP (ER-YFP-HDEL) displays reticulated fluorescence (B). YFP-PEX19A (D) and YFP-PEX19B (E) fluorescence patterns are neither punctate nor reticulated but resemble untagged YFP fluorescence (C), suggesting cytosolic localization. Each pair of images captures the same cells imaged through the middle (left column) or subcortical region (right column) of the cells. See S1 Fig for corresponding bright-field images. (F) Seedlings relying on YFP-PEX19A or YFP-PEX19B as the sole source of PEX19 respond to IBA similarly to wild-type seedlings. Error bars show standard deviations of mean 8-day-old light-grown root lengths (*n* ≥ 9). (G) Expression of YFP-PEX19B confers slight PTS2-processing defects. Protein extracted from 8-day-old light-grown seedlings was separated in triplicate using 10% PAGE and processed for immunoblotting with antibodies recognizing PEX19 or GFP (to detect YFP-PEX19; top and middle panels) and PMDH (bottom panels). Membranes were subsequently probed with α-HSC70 (loading control). The positions of the molecular mass markers (in kDa) are indicted at the left. PMDH is synthesized as a precursor (p) with a cleavable PTS2 signal that is processed into the mature (m) protein in the peroxisome.

To enable rescue of the *pex19a-1 pex19b-1* lethality, we transformed wild-type plants with constructs expressing N-terminally YFP-tagged PEX19A or PEX19B from the CaMV *35S* promoter (*35S*:*YFP-PEX19A* and *35S*:*YFP-PEX19B*). We found that both YFP-PEX19 proteins were localized similarly in a pattern that was neither punctate as exhibited by the YFP-ECH2 peroxisomal matrix protein [5] (Fig 6A) nor reticulated as exhibited by a marker of the ER lumen [55] (Fig 6B). Instead, YFP-PEX19A (Fig 6D; S1 Fig) and YFP-PEX19B (Fig 6E; S1 Fig) fluorescence resembled that of untagged cytosolic YFP (Fig 6C), again suggesting that *Arabidopsis* PEX19 is predominantly cytosolic, as previously reported [35] and consistent with the fractionation of PEX19 with soluble proteins (Fig 5).

To determine if the embryo lethality observed in the *pex19a-1 pex19b-1* double mutant was due to the loss of PEX19 function rather than unrelated mutations in these lines, we crossed wild type carrying the *35S*:*YFP-PEX19A* or *35S*:*YFP-PEX19B* construct to plants heterozygous for *pex19a-1* and homozygous for *pex19b-1* and sought plants homozygous for *pex19a-1*, *pex19b-1*, and the reporter transgene in the progeny from the cross. We obtained such lines, indicating that the lethality of *pex19a-1 pex19b-1* double mutant was restored by either YFP-PEX19A or YFP-PEX19B. Because these lines were viable, we assessed peroxisome function in the rescue lines. Both wild type and the *pex19a-1 pex19b-1* double mutant expressing YFP-PEX19A or YFP-PEX19B responded to IBA similarly to wild type (Fig 6F). However, we did observe a slight impairment in processing of PMDH in lines expressing YFP-PEX19B (Fig 6G), suggesting that expressing YFP-tagged PEX19 (unlike HA-tagged PEX19; Fig 4D) was slightly detrimental to peroxisome function. We concluded that the lethality of the *pex19a-1 pex19b-1* double mutant (Table 1) was indeed caused by loss of PEX19 function and that both *PEX19* isoforms encode functional PEX19.

### *pex19b-1* alters the physiological and molecular defects of *pex* mutants defective in docking complex peroxins

**Fig 7:**
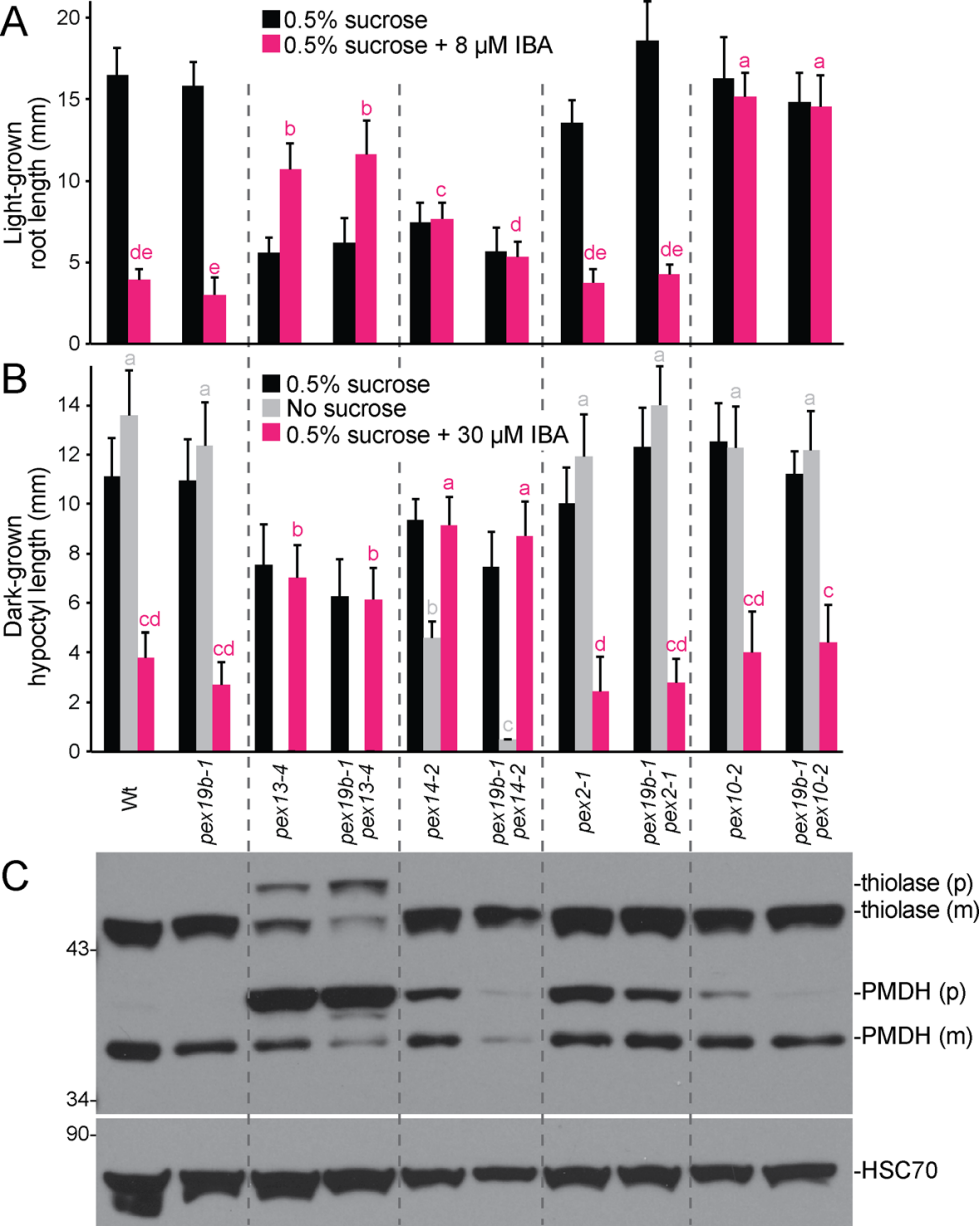
Reduced PEX19 function modulates defects of other peroxin mutants. (A) *pex19b-1* does not markedly alter the IBA responsiveness of *pex13-4*, *pex14-2*, *pex2-1*, or *pex10-2*. Error bars show standard deviations of mean 8-day-old light-grown root lengths of germinated seedlings (*n* ≥ 7). Different letters above bars indicate significantly different means (one-way ANOVA, *P* < 0.001). (B) *pex19b-1* exacerbates the sucrose dependence of dark-grown *pex14-2* seedlings. Error bars show standard deviations of mean 5-day-old hypocotyl lengths of germinated seedlings (*n* ≥ 5). No *pex13-4* or *pex19b-1 pex13-4* seeds germinated on medium lacking sucrose (*n* = 15). Different letters above bars indicate significantly different means (one-way ANOVA, *P* < 0.001). (C) *pex19b-1* worsens the PTS2-processing defect of light-grown *pex13-4* seedlings. Protein extracted from 8-day-old light-grown seedlings was separated using 10% PAGE and processed for immunoblotting with antibodies recognizing the indicated proteins. PMDH and thiolase are synthesized as precursors (p) with a cleavable PTS2 signals that are processed into the mature (m) proteins in the peroxisome.

To examine genetic interactions of PEX19 with other peroxins, we crossed *pex19b-1* to four mutants defective in various membrane peroxins: the docking complex peroxins (PEX13 and PEX14) and two RING-finger complex peroxins (PEX2 and PEX10). *pex13-4* is a partial loss-of-function missense allele altering the C-terminal domain of PEX13 [42], and *pex14-2* is a null allele disrupted by a T-DNA in the first exon of *PEX14* [43]. Both *pex13-4* and *pex14-2* exhibit sucrose dependence, IBA resistance, and PTS2-processing defects that reflect matrix protein import defects [42, 43]. *pex10-2* is a partial loss-of-function splicing allele that confers IBA resistance and PTS2 processing defects, and *pex2-1* is a partial loss-of-function missense allele with minor physiological defects but notable PTS2 processing defects [40]. We found that *pex19b-1* enhanced the PMDH and thiolase processing defects of *pex13-4* (Fig 7C) without significantly altering the severe physiological defects of this mutant (Fig 7A and 7B). In addition, *pex19b-1* worsened the sucrose dependence of *pex14-2* (Fig 7B) without notably altering PTS2-processing defects (Fig 7C). In contrast, *pex19b-1* did not significantly worsen the IBA responsiveness, sucrose dependence, or PTS2-processing defects of *pex2-1* or *pex10-2* (Fig 7).

## Discussion

PEX19 is an early-acting peroxin that binds and delivers a variety of PMPs to PEX3 for membrane insertion (reviewed in [17]). We found that *Arabidopsis* PEX19 accumulated most abundantly in aerial seedling tissues and was present at much reduced levels in mature rosette leaves (Fig 3), implying that PEX19 could be particularly important in early stages of plant development when peroxisomes are necessary to metabolize fatty acids and convert IBA to IAA. Despite a marked reduction in PEX19 levels in the *pex19b-1* mutant (Fig 3), we found that *Arabidopsis pex19a-1* and *pex19b-1* single mutants displayed wild-type β-oxidation phenotypes (Fig 4A and 4B). Similarly, reducing levels of *PEX19A* or *PEX19B* via RNAi does not impart notable β-oxidation defects [37]. Moreover, we found that both *pex19a-1* and *pex19b-1* processed PTS2 proteins normally and displayed wild-type levels and membrane association of tested PMPs (Figs 3–5). Therefore, we attempted to isolate a *pex19a-1 pex19b-1* double mutant, but double mutant seedlings were not recovered (Table 1). This lethality could be rescued by either YFP-PEX19A or YFP-PEX19B, indicating that the two PEX19 isoforms function redundantly and that the nearly undetectable amount of PEX19A remaining in the *pex19b-1* mutant is sufficient to provide PEX19 function in our growth conditions. Like the *pex19a-1 pex19b-1* double mutant, embryo lethality has been reported for null alleles of most membrane peroxins in *Arabidopsis* (reviewed in [1]), and *PEX19* mutations in the human peroxisome deficiency disease, Zellweger Syndrome, can result in death in infancy [66, 67].

PEX19 is farnesylated in yeast [23] and mammals [30]. Although farnesylation increases the strength of PEX19-PMP interactions [31, 32], farnesylation is not strictly required for PEX19 function in yeast or mammalian cells as overexpression of PEX19 derivatives that cannot be farnesylated rescues *pex19* mutant defects [68]. Using prenylation mutants, we observed that PEX19 is largely farnesylated in *Arabidopsis* (Fig 2). Like *era1-1*, the *Arabidopsis* farnesyltransferase β-subunit mutant, the yeast *ram1* farnesyltransferase β-subunit mutant accumulates unprenylated PEX19 [32]. Interestingly, yeast *ram1* mutants display reduced levels of several PMPs [32]. In contrast, the robust IBA responses of *era1-2* and the *plp-4* prenylation mutant (Fig 4A and 4B) and the normal levels of PMPs (Fig 4C) and PTS2 processing (Fig 4D) that we observed in these mutants suggests that farnesylation of *Arabidopsis* PEX19 is not essential for peroxisome biogenesis or function in our growth conditions, even when unfarnesylated PEX19 is present at wild-type levels (Fig 2C). Thus a functional role for PEX19 prenylation, which is implied by the evolutionary conservation of this modification (Fig 1A), is not revealed in our general physiological and molecular assays for peroxisome function.

Because reducing PEX19B levels did not seem to impact peroxisome function in an otherwise wild-type background, we constructed various double mutants with *pex19b-1* to assess peroxisome function in sensitized backgrounds. The *pex2-1* and *pex10-2* partial loss-of-function mutants display relatively minor peroxisome-related defects that are dramatically enhanced in a *pex2-1 pex10-2* double mutant [40]. In contrast to this enhancement, the *pex19b-1 pex2-1* and *pex19b-1 pex10-2* double mutant resembled the respective *pex2-1* and *pex10-2* single mutants in IBA resistance and sucrose independence (Fig 7), despite the documented interaction between PEX19 and PEX10 in *Arabidopsis* [35], yeast [69], and mammalian cells [31]. In contrast, *pex19b-1* worsened the PTS2-processing defect of the *pex13-4* partial loss-of-function allele and exacerbated the sucrose dependence of the *pex14-2* null allele (Fig 7). This double mutant analysis is consistent with the possibility that the early steps in matrix protein import carried out by the receptor-docking peroxins PEX13 and PEX14 are more sensitive to PEX19 levels than are the PEX5-recycling steps carried out by the PEX2 and PEX10 RING-finger peroxins. Alternatively, it is possible that the more severe initial defects of the *pex13* and *pex14* alleles used in these experiments rendered these mutants more sensitive to reduced PEX19B levels. Future experiments with weaker *pex13* [70] and *pex14* [71] alleles might resolve this question.

In summary, we found that *Arabidopsis* PEX19 is farnesylated and essential for embryonic viability and that *PEX19B* encodes the predominant PEX19 isoform in *Arabidopsis*. The reduced PEX19 levels in the *pex19b-1* mutant do not markedly impair peroxisome function in isolation but negatively impact peroxisome function in two mutants with existing defects. The mutants and reporters developed in this work will enable future examination of PEX19 function in plants.

## Supporting Information

S1 Fig YFP-PEX19 is mostly cytosolic. Cotyledon epidermal cells from 5-day-old light-grown seedlings carrying various YFP-tagged constructs were imaged using confocal microscopy. YFP directed to the peroxisome (YFP-ECH2) displays punctate fluorescence (A) and ER-directed YFP (ER-YFP-HDEL) displays reticulated fluorescence (B). YFP-PEX19A (D) and YFP-PEX19B (E) show fluorescence patterns that are neither punctate nor reticulated but that resemble untagged YFP fluorescence (C), suggesting cytosolic localization. For each construct, each row of images captures the same cells imaged through the middle (top row) or subcortical region (bottom row) of the cells. Columns show YFP fluorescence (left), bright field (middle), and merged images (right).
(PDF)

S1 Table PCR-based markers used for genotyping mutant alleles. (PDF)
