# Supplementary material for: The Early-Acting Peroxin PEX19 Is Redundantly Encoded, Farnesylated, and Essential for Viability in Arabidopsis thaliana
Source: PLoS One. 2016 Jan 29;11(1):e0148335. doi: 10.1371/journal.pone.0148335 (PMC4733102; doi:10.1371/journal.pone.0148335)
Supplement: S1 Fig — Cotyledon epidermal cells from 5-day-old light-grown seedlings carrying various YFP-tagged constructs were imaged using confocal microscopy. YFP directed to the peroxisome (YFP-ECH2) displays punctate fluorescence (A) and ER-directed YFP (ER-YFP-HDEL) displays reticulated fluorescence (B). YFP-PEX19A (D) and YFP-PEX19B (E) show fluorescence patterns that are neither punctate nor reticulated but that resemble untagged YFP fluorescence (C), suggesting cytosolic localization. For each construct, each row of images captures the same cells imaged through the middle (top row) or subcortical region (bottom row) of the cells. Columns show YFP fluorescence (left), bright field (middle), and merged images (right). (PDF) [file pone.0148335.s001.pdf]

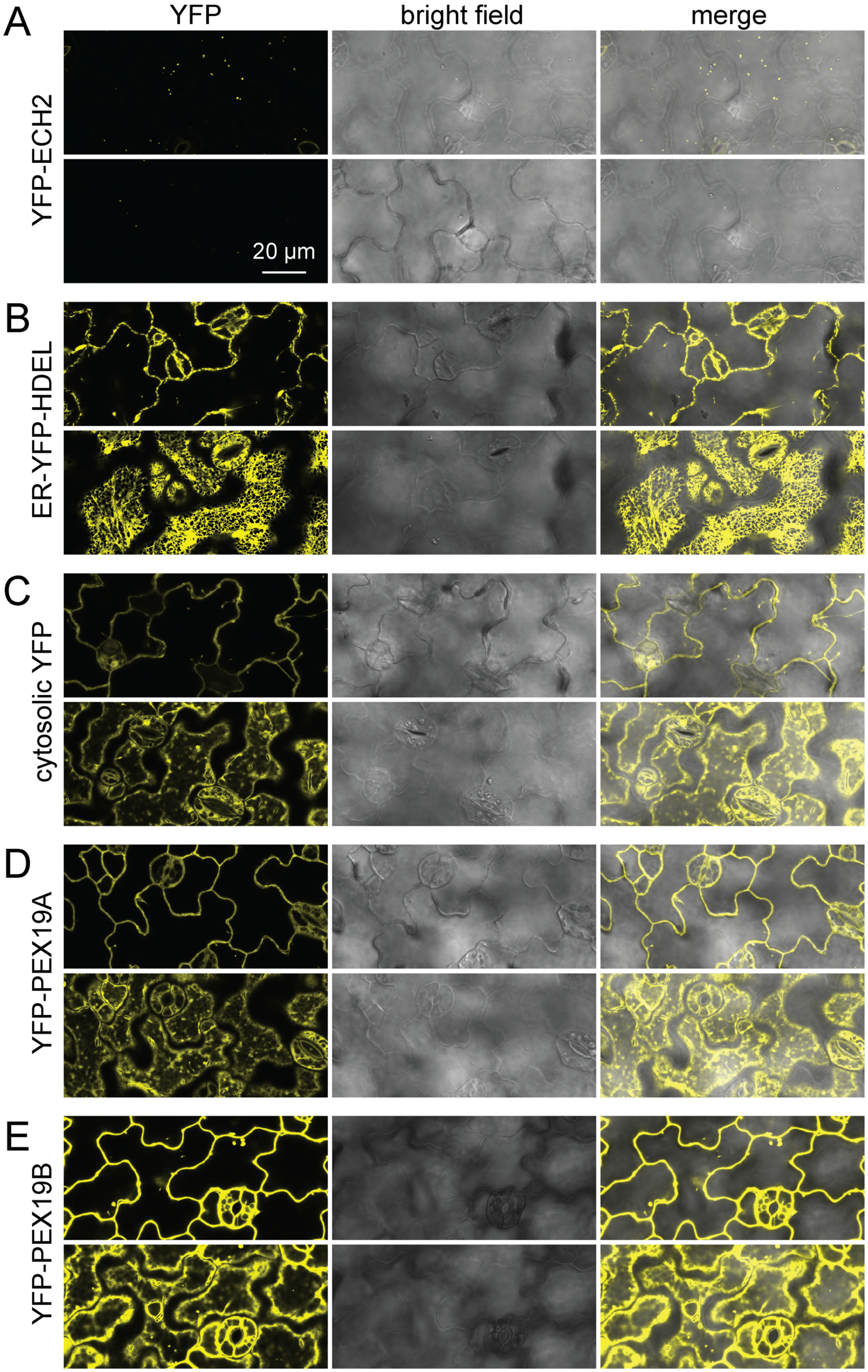

S1 Fig 1. YFP-PEX19 is mostly cytosolic.
Cotyledon epidermal cells from 5-day-old light-grown seedlings carrying various YFP-tagged constructs were imaged using confocal microscopy. YFP directed to the peroxisome (YFP-ECH2) displays punctate fluorescence (A) and ER-directed YFP (ER-YFP-HDEL) displays reticulated fluorescence (B). YFP-PEX19A (D) and YFP-PEX19B (E) show fluorescence patterns that are neither punctate nor reticulated but that resemble untagged YFP fluorescence (C), suggesting cytosolic localization. For each construct, each row of images captures the same cells imaged through the middle (top row) or subcortical region (bottom row) of the cells. Columns show YFP fluorescence (left), bright field (middle), and merged images (right).
