# Supplementary material for: The Early-Acting Peroxin PEX19 Is Redundantly Encoded, Farnesylated, and Essential for Viability in Arabidopsis thaliana
Source: PLoS One. 2016 Jan 29;11(1):e0148335. doi: 10.1371/journal.pone.0148335 (PMC4733102; doi:10.1371/journal.pone.0148335)
Supplement: S1 Table — (PDF) [file pone.0148335.s002.pdf]

**S1 Table. PCR-based markers used for genotyping mutant alleles.**

| Mutant (T-DNA insertion) | Accession number | Primer name (sequence) | Restriction enzyme | Product size (bp) | |
|---|---|---|---|---|---|
| | | | | Wt | Mutant |
| *era1-2* | *At5g40280* | ERA1-4 (GTTTGCTTTCTCATGTCGCTTGTCAGGGCT)<br>ERA1-5 (CCTTGGCTTTGTTACTGGATTCTTCATTC) | -- | 280 | none |
| | | upERA1 (AAAAGTCTGCATAACCGTAACC)<br>downERA1 (GGAAACCTTGAAAATAACAC) | -- | none | 410 |
| *ggb-3* (SALK_015072) | *At2g39550* | GGB-2 (GCGCCGAGAAAATGAAGACCCGAG)<br>LB1-SALK (CAAACCAGCGTGGACCGCTTGCTGCAACTC) | -- | none | ~600 |
| | | GGB-2 (GCGCCGAGAAAATGAAGACCCGAG)<br>GGB-1 (ATAAAATAAAATCAATGCCCCCAATG) | -- | 570 | none |
| *pex2-1* | *At1g79810* | PEX2-18 (TGCGTTGCCTCCGTTGGTGGTCAG)<br>PEX2-DpnII (CATACAGACCTGCTCAGAATCACCCGAT) | *Dpn*II | 65, 28 | 93 |
| *pex7-2* | *At1g29260* | PEX7-N8 (CTCCAGAAGCAGAAGCAAACACATCAC)<br>PEX7-Tsp45I (CAATCCCACGCGGCGCGATTCGTTTGTCA) | *Tsp*45I | 141, 29 | 170 |
| *pex10-2* | *At2g26350* | PEX10-11 (CGTTGAAGTTGAATCGGAGGTAGAC)<br>PEX10-PstI (AATATAGTTTTGGTATTGTTCCTGCA) | *Pst*I | 91, 28 | 119 |
| *pex13-4* | *At3g07560* | PEX13-19 (CTTATAGATCAAAACACACAGGCCTTTCACATG)<br>PEX13-HinfI (AAGCATACGCAGTACAAATCTTGCTGATT) | *Hin*fI | 180, 31 | 211 |
| *pex14-2* (SALK_007441) | *At5g62810* | PED2-9 (GCTTGCTGAACCTCATTAGCAGGCTTAGTAGCC)<br>LB1-SALK (CAAACCAGCGTGGACCGCTTGCTGCAACTC) | -- | none | ~600 |
| | | PED2-9 (GCTTGCTGAACCTCATTAGCAGGCTTAGTAGCC)<br>PED2-1 (CATCCTCATCATCTCTCATCAT) | -- | 460 | none |
| *pex19a-1* (SALK_020100) | *At3g03490* | PEX19A-9 (CAGTTTCCCTAGTTTGTTCCC)<br>LB1-SALK (CAAACCAGCGTGGACCGCTTGCTGCAACTC) | -- | none | ~300 |
| | | PEX19A-9 (CAGTTTCCCTAGTTTGTTCCC)<br>PEX19-4S (GGATCGTGTACTTTGGCTTCACCGCAAC) | -- | 777 | none |
| *pex19b-1* (SAIL_76_C06) | *At5g17550* | PEX19B-1 (AAAAATGGGCTTACGACACAACAC)<br>LB3-SAIL (TAGCATCTGAATTTCATAACCAATCTCGATACAC) | -- | none | ~500 |
| | | PEX19B-1 (AAAAATGGGCTTACGACACAACAC)<br>PEX19B-8 (TCCCACCAAAAACATAACAGAACCTC) | -- | 506 | none |
| *plp-4* (GABI-KAT 386C07) | *At3g59380* | PLP-3 (GAAGCTGACGTCTTTAACAATTCC)<br>LB1-GABI (ATATTGACCATCATACTCATTGC) | -- | none | ~500 |
| | | PLP-3 (GAAGCTGACGTCTTTAACAATTCC)<br>PLPcDNA-2 (TCAAATTGCTGCCACTGTAATCTTGC) | -- | 566 | none |
